# The use of zootherapeutics in folk veterinary medicine in the district of Cubati, Paraíba State, Brazil

**DOI:** 10.1186/1746-4269-3-32

**Published:** 2007-09-07

**Authors:** Raynner RD Barboza, Wedson de MS Souto, José da S Mourão

**Affiliations:** 1 St. Geraldo Soares de Almeida, nr. 111, Catolé, P.O. 58105-041, Campina Grande-PB, Brazil; 2 Curso de Licenciatura e Bacharelado em Ciências Biológicas, Universidade Estadual da Paraíba, Av. das Baraúnas, nr. 351/Campus Universitário, Bodocongó, P.O. 58109-753, Campina Grande-PB, Brazil; 3 Departamento de Biologia, Universidade Estadual da Paraíba, Av. das Baraúnas, nr. 351/Campus Universitário, Bodocongó, P.O. 58109-753, Campina Grande-PB, Brazil

## Abstract

**Background:**

The present work addresses the use of zootherapy in folk veterinary medicine (ethnoveterinary) by the residents of the municipal district of Cubati, microregion of Seridó, Paraíba State, Brazil. It sought to identify the principal animals used as medicinal sources for zootherapeutics and to contribute to the preservation and sustainability of this traditional knowledge.

**Methods:**

Field research was undertaken on a weekly or biweekly basis during the period November, 2006, to January, 2007. Free, semi-structured, and open interviews were made with local residents of the municipal district of Cubati (in both urban and rural settings) as well as with venders in public markets. A total of 25 individuals of both sexes were interviewed (with ages varying from 26 to 78 years) although only 16 were finally chosen as informants as these people demonstrated the greatest degree of knowledge concerning zootherapeutics. Graphs and percentages were generated using Microsoft^© ^Excel 2007 software, and the species were identified by photographic registration and subsequent bibliographical surveys.

**Results:**

Mammals constitute the main medicinal zootherapeutic source for folk veterinary medicines in the studied area, both in terms of the total number of species used and the frequency of their citation. Sheep (*Ovis aries*), pigs (*Sus scrofa*), cattle (*Bos taurus*), and foxes (*Cerdocyon thous*) were mentioned by 62.5, 43.75, 37.5, and 31.25% of the informants, respectively, as being used in folk veterinary medicine. Additionally, chameleons (*Iguana iguana*), chickens (*Gallus domesticus*), and rattlesnakes (*Crotalus durissus*) were mentioned by 75, 43.75, and 31.25% of the informants, respectively. Relatively simple animal illnesses, such as furuncles, or injuries resulting from embedded thorns or skin eruptions are responsible for the largest number of zootherapeutic treatment, while, diseases of greater complexity, such as rabies and brucellosis, were not even mentioned. Fat from various animals constituted the most frequently cited resource used for its medicinal-veterinary properties.

**Conclusion:**

The examination of folk knowledge and health practices allows a better understanding of human interactions with their local environment, and aids in the formulation of appropriate strategies for natural resource conservation.

## Background

*"Formerly we had to get our medicines from nature. There were neither doctors nor veterinarians or nothing" *(a 74 year old man)

Humans have utilized animals to produce drugs for treating illnesses and injuries since ancient times [1,2]. However, it can be assumed that concern about animal health only originated after the domestication of formerly wild species for use in transportation, agriculture, or as direct food sources. Traditional folk knowledge concerning the use of natural medicines is now present in every known culture.

In the last few years, folk knowledge of traditional populations has been recognized as being of significant importance in several areas of the natural sciences – as the study of ethnoscience – with the prefix "ethno" referring to the knowledge systems of traditional cultures [3]. The study of ethnosciences began to expand beginning in the middle of the last century, and workers in several areas began to incorporate the term "ethno" into their scientific repertoire, such as in ethnobotany [4,5]; ethnozoology [5], and ethnoveterinary [6,7]. Ethnoscientific knowledge is transmitted almost exclusively through an oral tradition in essentially all societies examined [1,2,7-9].

The present work examines the ethnoscientific knowledge linked to the use of animals (zootherapeutics) as sources of medicines in folk veterinary medicine (ethnoveterinary). This term was firstly used by McCorkle in the mid 1980's to designate the "people's knowledge, abilities, methods, practices and beliefs concerning animal health care". Mathias [6] emphasized that this knowledge is acquired by communities over many years on the basis of exhaustive trial and error. This same author affirms that the current scenario of rapid cultural changes in communities throughout the world [mainly due to globalization] is putting acquired ethnoveterinary knowledge at risk, and in many places there now exists a mix of traditional knowledge and modern veterinary medicine. In truth, both of these medicinal practices have their limitations and can complement each other; for in certain circumstances traditional knowledge can be perfectly applied, while in other situations modern medicinal practices are more effective [6]. It should be pointed out that folk veterinary medicine has been the starting point for the discovery of many drugs now used in modern animal medicine [2].

Ethnoveterinary is a valuable tool with great potential, if used in a sustainable manner [10]. In poorly developed areas, agricultural activities are responsible for a large percentage of the local income (as in the microregion of Seridó, Paraíba State, Brazil, examined here) [11] and folk veterinary medicine constitutes an important alternative to modern veterinary medicine, especially in terms of its cost.

However, very little ethnoveterinary knowledge has been documented in Brazil in spite of its great local importance. In many areas (such as the interior of Paraíba) this folk knowledge is rapidly disappearing – with traditional medicine being set aside in favor of modern medical practices, and veterinary drugstores are now frequently found in formerly isolated areas.

The decline of popular veterinary medicine in different parts of the world has been described by authors such as Jacob et al. [12] and Mathias [6]. The objective of this work, therefore, was to identify the animal sources of ethnoveterinary medicines in the municipal district of Cubati, Paraíba State, and the main diseases treated using these zootherapeutics, as well as to contribute to the preservation of folk knowledge that is rapidly being lost.

## Methods

### Profile of the Community Examined

The State of Paraíba occupies 56,341 km^2 ^[13] and has a population of approximately 3,443,825 inhabitants, of which 1,937,738 are black, 1,467,260 are white, while 38,827 belong to other ethnicities [13].

**Figure 1:**
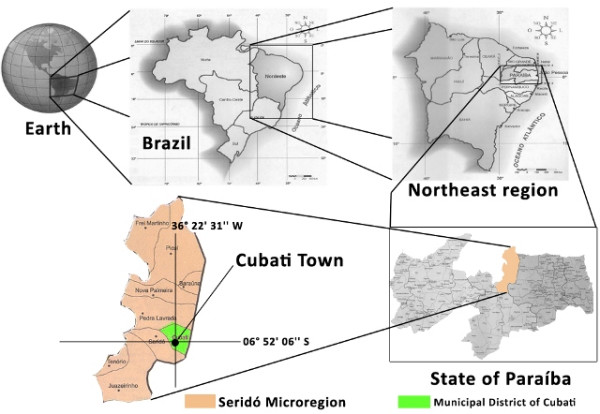
**Map of the Localization of Cubati Town**. Adapted of Rodriguez et al. [11]

The municipal district of Cubati covers an area of 161.3 km^2 ^and is located in the eastern microregion of Seridó, Paraíba [13,14], at the approximate geographical coordinates 06° 52 ' 06 " S and 36° 22 ' 31 " W [15] (see Figure 1). The local vegetation is composed of semideciduous and deciduous forests characteristic of this semi-arid region. Average annual rainfall totals 500 mm, with an annual average temperature of 26°C [11,16], and the region is within the "drought polygon" of Brazil (an area that extents from northern Minas Gerais State to almost the entire northeastern part of that country) [17]. The total population of the municipal district is estimated to be 6,456 [18], with a Human Development Index (IDH) of 0.591 (medium level development) [13]. Approximately 4,030 inhabitants live in the urban zone, with the remaining population occupying rural areas; the average monthly per capita income in R$ 87.22 (approximately US$45.00) [13]. Subsistence agriculture (especially cassava, corn, beans) and livestock husbandry, especially of goats (1,500 animals), sheep (1,000), and cattle (1,500), constitute the main economical activities in the municipal district [19-21]. The traditional veterinarian knowledge of the inhabitants of Cubati is almost exclusively held by individuals linked to agricultural activities, and this information is generally transmitted orally from generation to generation.

### Procedures

Field research was undertaken during weekly or biweekly excursions during the period from November, 2006, to January, 2007. Free, semi-structured, and open interviews were conducted with local residents of the municipal district of Cubati (in both urban and rural zones) in the communities of Portal, Praia Nova, and Lajedo do Angico, as well as with vendors in public markets at these localities. Informants were selected starting from a wide pool of individuals casually encountered in the communities, as well as based on the criterion of "local specialists" – people recognized within the community itself as having exceptional knowledge about zootherapeutics [22].

A total of 25 individuals of both sexes with ages varying from 26 to 78 years were interviewed, although only 16 were finally chosen as informants based on their specialized knowledge. Using the principals outlined by Andrade and Costa Neto [23], the interviewees were provided with an initial explanation of the objectives of the research, and once their approval of the project was obtained, they were asked to answer questions on the following subjects: which animals (zootherapeutics) were used for the production of medicines; what are the purposes of those medicines; and how are they used. We always attempted to establish a greater rapport with the local residents by using local terminology and expressions during the interviews. The names of the zootherapeutic animals, and the symptoms and/or treated diseases were recording according to the terminology used by the interviewees. We adopted the use of written questionnaires in which the interviewers noted the information provided by the informants, taking the precaution of repeating and confirming the information provided more than once during the interview, as recommended by Barboza [24] and Costa Neto [25]. The interviews lasted from 20 minutes to a little more than an hour. The transcribed interviews are stored at the Laboratory of Zoology of the State University of Paraíba. Graphs and percentages were produced using Microsoft^© ^Excel 2007 software.

### Species identification

Specimens of the wild animals cited were digitally photographed for more precise identification, and the animals were then subsequently released to their natural habitats. Species identification was accomplished using bibliographic information, including Albuquerque et al. [26], Alves et al. [27], IBAMA [28], Brazil [29], Brazilian Ornithological Committee Registrations (CBRO) [30], Rodrigues [31], Rodrigues [32], Oliveira et al. [33], Silva et al. [34], Silveira [35], and Sousa and Gonçalves [36].

## Results and Discussion

Interviews with key-informants in the municipal district of Cubati revealed that popular medicinal-veterinarian knowledge was transmitted from generation to generation:

*"I got it from my father, and my father got it from my grandfather" *(a 66 year old man).

*"It works like this: the oldest [parents, grandparents among others] tried different things and what they learned they passed on to us" *(woman, 70).

Experiences transmitted through generations are known by memes – recognizable fragments of cultural information that are transferred from person to person within a culture [37]. Cognitive inheritance is mentioned in several works (whether directly, in the sense of a "meme", or indirectly), such as Araújo et al. [38], Costa Neto [25], Costa Neto and Oliveira [1], Jacob et al. [12], Lima and Vasconcelos [39], and Mourão et al. [40]. It should be point out that none of the zootherapeutics mentioned in the interviews were derived from animals threatened with extinction as listed by the Brazilian Environmental Ministry [41].

Brazilian laws prohibit hunting wild animals, such as the rattlesnakes (*Crotalus durissus*) and foxes (*Cerdocyon thous*) reported as used here, and it will be important to make the local residents aware of the necessity of preserving the animals that inhabit the *caatinga *biome and to avoid over-exploiting these otherwise renewable natural resources beyond their recovery capacities.

*"We cannot capture armadillos or foxes because the agents from IBAMA prohibit it. The fines are too high (...). Formerly we had a lot of armadillos to eat and to make medicine. Today only a few remain. (a 66 year old man replying to an informal question regarding the possibility of capturing a fox for photographic registration)*.

This does not imply that natural resources should not be used, however, for with well-managed conservation efforts these animals could continue to be viable sources of low-cost veterinary pharmaceutical substances for the local population within the concept of the *World Conservation Union *(IUCN).

*"Sustainable use (whether extractive or non-extractive) is a dynamic process in which an effort is made to maintain biodiversity and to reinforce ecological and socio-ecological services, recognizing that the larger the patrimony and the more the government participates, the greater will be the probability of reaching these objectives for present and future generations" *[42].

The animal model of disease allows the comparison of human and animal illnesses [43] and relies on pharmaceuticals or treatments being tested in animals before their use by humans. Currently, a animal model is being used with guinea-pigs in the development of a vaccine against human rheumatic fever caused by the bacteria *Streptococcus pyogenes *[44]. Interestingly, the local inhabitants that still practice ethnoveterinary medicine are empirically using a mirror-image model, which could be called the "*human model for diseases in animals*". Starting from the principle of similarity, many of the zootherapeutics used by the residents of the Cubati community for treating human diseases are also used as treatments for animal diseases:

*"If it works for men, it works for animals also" *(a 74 year old man).

*"For treating puncture wounds in animals ****it is the same way as for us****. Just place the chameleon skin powder mixed with food oil on top of the wound, and after 2 days it is already better" *(a 38 year old man).

*"The fat from a teju *(a large lizard)*or a peba *(armadillo) *is a good medicine for illnesses [wounds] ****for us as it is for the animals****" *(a 74 year old woman).

**Figure 2:**
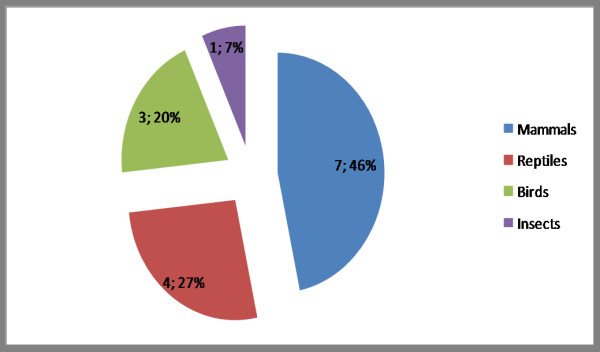
Percentage of the groups used with zootherapics in the veterinary medicine in Cubati in relation of the mentioned species type. Probably mammals and reptiles stand out due the characteristics of the local fauna and the agricultural activity.

In the present work, a disease is considered any type of anatomical or physiologic alteration of an animal as compared to its normal state. Among the fifteen zootherapeutics cited (Table 1), there is a clear predominance of mammalian origin – seven species (including the man) (ca. 46%), followed by reptiles with four species, or approximately 27% (see Figure 2).

**Table 1:** Animals used in the Popular Veterinary Medicine in Cubati – PB

Animal		Percentage of Citation	Used Part	Indications
			
Popular Vernacular	Species			
***Mammals***				

Fox	*Cerdocyon thous*	31.25% (n = 5)	Fat	Uterus prolapse
Ram	*Ovis aries*	62.5% (n = 10)	Castrated Ram Suet	Arthritis, Bovine Gangrenous Coryza (hollow of the ox horny), rheumatism, woundy, edema, wounds, pointed stakes, fractures, swellings, furunculosis.
Cattle	*Bos taurus*	37.5% (n = 6)	Butter of the milk cream	Pits, mastitis
			Butter of the curdled milk cream	peri-oculars Irritations
			Milk	Fractures
			Suet	Bovine Gangrenous Coryza (hollow of the ox horny)
Pig	*Sus scrofa*	43.75% (n = 7)	Fat	Pits, mastitis, burns, wounds, furunculosis.
			Fat of the pig scrotum	Pits, pointed stakes, fractures, furunculosis, mastitis, torsion
Male goat	*Capra hircus*	12.5% (n = 2)	Castrated Male Goat Fat	General wounds
			Feces	pointed stakes
Armadillo- Peba	*Euphractus sexcinctus*	18.75% (n = 3)	Fat	Pits, pointed stakes, wounds, furunculosis
*Humans*	*Homo sapiens*	18.75% (n = 3)	Urine	Intoxication of the cattle when the ones feed of cassava (*Manihot utilissima Pohl*.)

***Reptiles***				

Rattlesnake	*Crotalus durissus*	31.25% (n = 5)	Fat	Wounds, Arthritis
Chameleon	*Iguana iguana*	75% (n = 12)	Leather	Pits, pointed stakes, furunculosis.
			Fat	Pointed stakes, General wounds
Turtle of water	*Phrynops spp*.	25% (n = 4)	Fat	Swellings
Lizard-Teju	*Tupinambis merianae*.	18.75% (n = 3)	Fat	Pits, pointed stake, wounds, furunculosis

***Birds***				

Hens	*Gallus domesticus*	43.75% (n = 7)	Fat	Mastitis, furunculosis
			Egg	Uterus prolapse
			Feces	Mastitis
			Gizzard skin	Burns
Peru	*Meleagris gallopavo*	18.75% (n = 3)	Fat	Furunculosis
Quail	*Nothura maculosa cearensis*	18.75% (n = 3)	feathers	Snakes bites

***Insects***				

Hornet-mestizo	*Polistes canadensis*	12.5% (n = 2)	Sting	Cattle pits

**Figure 3:**
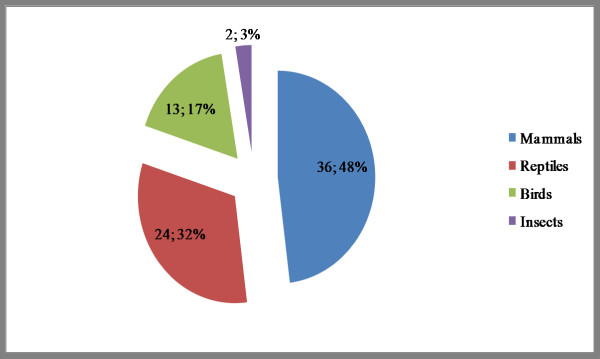
Groups representation in relation to the species citation frequency. For instance, the 7 found zootherapics mammals species are mentioned 36 times, or 48% of the total zootherapics citations. Reptiles and Mammals represents 80% of the citations.

In a sense, these results could be expected due to the agricultural vocation of the region and its local fauna. For example, pigs (*Sus scrofa*), cows (*Bos taurus*), and sheep (*Ovis aries*) are commonly raised in the area, while rattlesnakes (*Crotalus durissus*), chameleons (*Iguana iguana*), and the *teju *lizards (*Tupinambis merianae*) are reptiles frequently encountered in the local *caatinga *vegetation. Mammalian species were mentioned 36 times (48% of the total number of citations), while reptiles were cited 24 times (32%) (see Figure 3).

As can be seen in Table 1, a large number of folk veterinarian medicines are produced from animal fats, and these are usually applied on wounds, furuncles, sores, or embedded thorns. Furuncles represent infections of the hair follicles or obstructions of the oil glands, mainly caused by *Staphylococcus sp*., and involve subcutaneous cellular tissues [45]; while sores have other origins, especially those related to allergic reactions or insect bites and stings. Chameleons (*Iguana iguana*), mentioned by 75% of the interviewees (N = 12), produces a type of body fat that aids in wound healing and is used to promote the natural expulsion of foreign bodies from cows, horses, and chickens.

*"When there is a thorn embedded in a cow just put chameleon fat on top of it. After about 3 days it pulls the thorn out" *(a 70 year old woman).

*"Chameleon fat is a very good medicine. I use it for removing thorns from cows and donkeys" *(a 66 year old man).

Mastitis, an inflammation of the mammary glands, is the most common and expensive disease of dairy cows in most of the world [46] (affecting other types of animals as well). According to the interviewees, this disease is treating in cows by using butter, or fat derived from pigs and chickens (see Table 1 and Table 2). The suet from castrated sheep (*Ovis aries*) was mentioned by 62.5% of the interviewees (N = 10), who cited its use for treating several health problems in animals, including problems with their horns, wounds, embedded thorns, and furuncles, among others. Table 3 lists the popular vernacular names for diseases or animal illnesses, with their respective technical terms.

**Table 2:** Preparation and application manners of the cited zootherapics in the text

***Mammals***	
**Ram **(*Ovis aries*) *see figure 5*	After the animal slaughter, it takes out the fatty tissue below the skin – the "suet" – which is referred as medically important for humans and animals. After the extraction, it is putted down to dry in the sun for approximately two days or until it acquires a hard consistence. For a better conservation, it should be kept in a dry place, inside a glass or plastic recipients. In animals, for arthritis's (swelling joints), rheumatism's (hard joints), woundy's, edema's (big swell), wounds', pointed stakes', fractures', swellings' and furunculosis' cases, the application is identical. Melts the suet, applies it on the affected area and when necessary just make a curative. In relation to the Bovine Gangrenous Coryza (hollow of the ox horny), just saw the point of the ox horny, and fill its interior with the suet. This is just a palliative way. It should be pointed out that just the castrated ram suet has the medicinal properties.

**Mele Goat **(*Capra hircus*) *see figure 6*	After the fatty tissue has been removed, melts it in order to obtain a sharp fat (process known by the local inhabitants as "condensation"). The fat is conserved in a glass or plastic recipient and whenever need it is necessary to melt again, once it rigidifies inside the recipient after a certain period of time. It's used in the local veterinary medicine to treat wounds in animals, mainly in the cattle, pigs, equine and birds. It should be applied passing the fat on the wound and making a curative after. Just the fat of the castrated male goat is medicinal.

**Cattle **(*Bos taurus*) *see figure 7*	The suet, extracted, stored and applied exactly the same way it does on the castrated ram (*Ovis aries*) is used for the treatment of the Bovine Gangrenous Coryza (hollow of the ox horny). The home-made butter produced from the cow milk cream is used to cure pits and mastitis. In the first case, just pass the butter on the pit until it heals and disappear; on mastitis the butter is applied on the mammas by massaging them. The treatment proceeds up the total cure. Another butter type, prepared from the cattle curdled milk cream, is used for the treatment of peri-oculars irritations (in any animal, according to the informers). The application is quite simple. When the animal has an irritation near the eyes, just pass this butter daily, until obtain the cure. The cattle milk, associated with the wormseed (*Chenopodium ambrosioides*) is used for the treatment of animals' fracture. From these two components it is prepared a kind of juice and given to the fractured animal to drink twice a day and, according to one of the reports, in about two weeks the animal is practically cured.

**Humans **(*Homo sapiens*)	The humans' urine is used for cattle detoxication after the feed of cassava (*Manihot utilissima Pohl*.). The preparation is diluting a cup of human urine in 3 liters of water, mixing well and giving for the cattle to drink after the plant ingestion. Such statement resembles the urinotherapy used by humans for several treatments.

**Peba **(*Euphractus sexcinctus*) *see figure 8*	The Peba is a type of armadillo known in a large part of the Northeast region, used gastronomically and medically. In Cubati's popular veterinary medicine the peba's fat is an important tool for the treatment of pits, pointed stakes, wounds and furunculosis in any animal. In all the cases, this fat is applied daily on the illness attacked area until its total cicatrization. In pointed stakes' cases, the application should persist until the complete exit of the strange body. The extraction and conservation of the Euphractus sexcinctus fat are made the same way that does on the Male goat (*Capra hircus*).

**Pig **(*Sus scrofa*) *see figure 9*	According to the informers, the pig's fat is an excellent medicinal-veterinary resource. It is used for the treatment of pits, furuncles, wounds, burns and mastitis. In the first four cases, it is applied on the illness, once a day, until the cicatrization be completed. For the mastitis, in any mammal, emphasizing the bovine, bovid and sheep, the pig's fat is applied on the mammary glands in the same way that does with the cattle curdled milk cream, previously mentioned. Specifically the fat of the pig scrotum is useful to cure and/or treat pits, furuncles, pointed stakes, fractures and torsions, in any animal. For pits or furuncles this fat should be applied once a day, until the total cicatrization. In pointed stakes, the fat of the pig scrotum is applied once a day too, until that strange body is expelled. In fractured areas or with torsions, is applied on the affected area, always making massage for a better result.

**Fox **(*Cerdocyon thous*) *see figure 10*	The fat of the fox (*Cerdocyon thous*) is used by the local residents for the treatment of the uterus prolapse, "mother's body" as they used to call. This problem refers to the animal's uterus extravasating after give birth. When this occurs, the fat of the fox is passed on the uterus after been washed in clean water. After the application, the residents used to place back the exposed part of the uterus carefully inside the animal. Such technique is just dominated by a few residents. It should be pointed out that the uterus should still be linked to the animal for the procedure to works. The fat of the fox is extracted the same way that the male goat.

***Reptiles***	

**Rattlesnake **(*Crotalus durissus*) *see figure 11*	The fat of the rattlesnake follows the same extraction procedure and conservation of the other mentioned animals. Such for arthritis and wounds it is applied on the sicken area until the cure is obtained. It was not mentioned a daily frequency of application. According to the informers, for any animal type, besides for the man, this fat is useful.

**Chameleon **(*Iguana iguana*) *see figure 12*	The chameleon's leather is used for treating pits, furuncles and pointed stakes' removing, such in animals and humans. In the first two cases, after the leather been extracted, it is toasted, triturated and the powder, mixed with food oil. After that is passed daily on the harmed area, until obtaining the cure. For the pointed stakes removing, the leather of the chameleon is placed on the area where the pointed stake is. The method of utilization is tying the harm area with a cloth and, after some hours, takes out the cloth pulling the leather of the chameleon in order to expel the pointed stake. The chameleon's fat (*Iguana iguana*) is extracted and prepared following the same procedure used with the male goat (*Capra hircus*). It is useful for the treatment of wounds and pointed stakes' removing. It's used daily until the total cicatrization (for wounds) or removal of the strange body (pointed stakes).

**Turtle of water **(*Phrynops spp*.) *see figure 13*	In animals, the fat of the turtle of water (*Phrynops spp*.) is used for treatment of swells. The application is on the swollen area during some days and, in a few days the harmed area is back to normal.

**Teju **(*Tupinambis merianae*) *see figure 14*	The teju's fat is used and applied daily in the treatment of pits, wounds, furuncles and pointed stakes. According to the informers, the use of that fat is made until the total cicatrization of the affected area or the strange body (pointed stake) removal. The extraction and conservation follows the same procedures mentioned previously for the zootherapics animals.

**Birds**	

**Hen **(*Gallus domesticus*) *see figure 15*	The chicken's fat (see the extraction and preparation of the male goat fat, *Capra hircus*, as example) is used for mastitis's treatment and furuncles in animals. For the first illness, it should pass the fat and make massage on the mamma daily; in the second case, it's enough to place the chicken fat on the area in order to control the secretion totally and consequently scars the furuncle. The chicken egg is used for uterus prolapse, being applied the same way that the fox fat (*Cerdocyon thous*). The egg is applied mixed in the exposed uterus. The chicken feces were indicating for the treatment of the mastitis (rocky nipples). According to some reports fresh chicken feces is passed on the inflamed mamas and leave them dry naturally. This procedure can be repeated as many times as possible until they return to normal conditions. The "skin" of the chicken gizzard was indicated for the treatment of animal's burns. The procedure is taking that "skin" from the gizzard, let it dry on the sun, triturated and the powder is placed on the burn.

**Peru **(*Meleagris gallopavo*) *see figure 16*	The peru's fat (see the extraction and preparation of the male goat's fat, *Capra hircus*, as example for procedure) was mentioned as useful for treatment of animals' furuncles. Follows the same method of application of the chicken's fat for this purpose.

**Quail **(*Nothura maculosa cearensis*) *see figure 17*	It was reported that the quail's feather, after being toasted and smashed into a powder, is useful in a tea preparation with antiophidic properties, given to the animals to drink (mainly the cattle) after bites of snakes.

**Insects**	

**Hornet-mestizo **(*Polistes canadensis*) *see figure 18*	The sting of this animal is used on cattle's pits in order to have a fast inflammation, followed by a quickly heal.

**Table 3:** Animals' diseases or illnesses popular names comparison, according to the inhabitants of Cubati, with the respective technical terms

**Popular Vernacular**	**Technical Vernacular**
Swelling joints	Arthritis
Pits	Pits
Hollow of the ox horny	Bovine Gangrenous Coryza (Horns drill)
Big swell	Edema
Pointed stake	Thorns
Woundy	Wounds
Wounds	Wounds or Cuts
Fractures	Fractures
Furunculosis	Furunculosis
Swellings	Swellings
Intoxication	Alimentary intoxication
Irritation near of the eye	Peri-ocular Irritation
"Rocky nipples"	Mastitis
Snake bites	Snake bites
"Mother's body"	Uterus prolapse
Burns	Burns
Hard joints	Rheumatism
Torsions	Torsions

**Note: **The technical terms were obtained comparing the symptoms supplied by the residents of the studied area with Andrei [51] and Kelly [52]

The frequent use of sheep suet as therapeutic resource for treating animal diseases may be related to the regional abundance of this animal. The reason why the suet must come from a castrated animal was explained in different ways by different individuals:

*"Because un-castrated sheep don't have fat, do they? But a castrated sheep does. It is thicker" *(a 74 year old man).

*"Because it works for medicine; because when it melts it becomes a fine oil, while the suet of a normal sheep becomes a thick oil when it melts" *(man, 78 years). The use of human urine was mentioned by 18.75% (N = 3) of the interviewees as a therapeutic agent. Cattle poisoned by feeding on cassava (Manihot utilissima Pohl.) are forced to drink it. The preparation is quite simple, requiring only mixing a cup of urine in 3 liters of water. The confirmed use of this treatment indicates that humans, too, can be considered a zootherapeutic resource.

*"If a cow eats cassava it has to be given people's urine to drink as quickly as possible, because it could die quickly" *(a 26 year old man).

Medicines prepared by the local residents were observed to have widespread applications, often serving not just for a single species, but for many of the animals that they raise. It was noticed during the visits and interviews that certain pathologies such as rabies (*Lyssavirus*) [47], brucellosis (*Brucella abortus*) [48], and bovine babesiosis (*Babesia bovis, Babesia bigemina*, and *Anaplasma marginale*) [49] do not have treatments based on the zootherapy, and these diseases must be treated with commercial pharmaceuticals or phytotherapeutics. Concerns about disease prevention or vaccination were scarcely mentioned, except concerning bovine foot-and-mouth disease (*Aphthovirus*) – as there is an annual governmental vaccination program and the cattlemen are obligated to vaccinate their herds.

**Figure 4:**
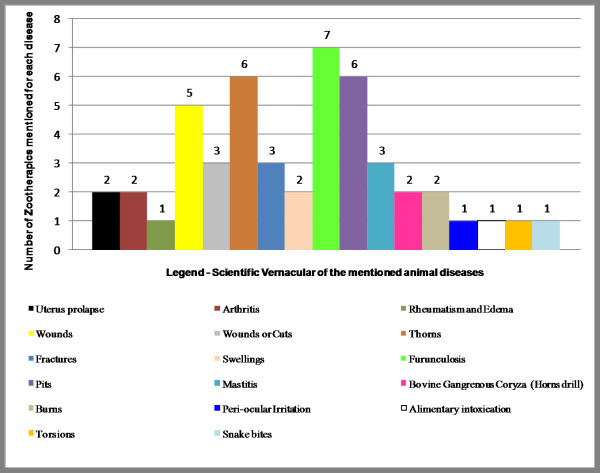
Comparison of the zootherapic number for each mentioned disease.

In terms of the number of different kinds of zootherapeutics used to treat a given illness, 7 folk medicines were mentioned for treating furuncles, followed by 6 zootherapeutics used in the treatment of embedded thorns and sores (not furuncles); only single zootherapeutic uses were mentioned as treatments or cures for rheumatism, edemas, peri-ocular irritations, food intoxication, torsions and snake bites (see Figure 4).

Some of the animals used to prepare ethnoveterinary medicines are wild native species and future studies will be needed to investigate the sustainable use and conservation of these species.

## Conclusion

The residents of the municipal district of Cubati (especially those economically dependent on agricultural activities) demonstrated a wide knowledge of natural products used as medicinal resources for themselves as for their domestic animals, and zootherapy plays a principal role in the prevention or cure of illnesses in that region.

The inhabitants of Cubati who deal with ethnoveterinary medicine often employ zootherapeutics in treating similar ailments that affect their domestic animals that are otherwise used to treat human diseases or illnesses, following an intuitive similarity between humans and other mammals.

*Mammals *supply the largest number of veterinary medicines in terms of their citation frequencies, followed by *reptiles*, *birds *and, infrequently, *insects*. However, only relatively simple to moderately complex illnesses or diseases are treated with zootherapeutics. The simplest infirmities, such as furuncles, embedded thorns, sores, and wounds, had the largest number of zootherapeutic treatments cited (seven, six, six, and five, respectively), while moderately complex medical problems, such as uterine prolapse, arthritis, rheumatism, and bovine gangrenous coryza had few zootherapeutic treatments or cures (with two, two, one, and two treatments cited, respectively). The preservation of popular medicinal knowledge is important to enhance our understanding of the relationships between society and nature, and to elaborate more effective strategies for conserving natural resources.

**Figure 5:**
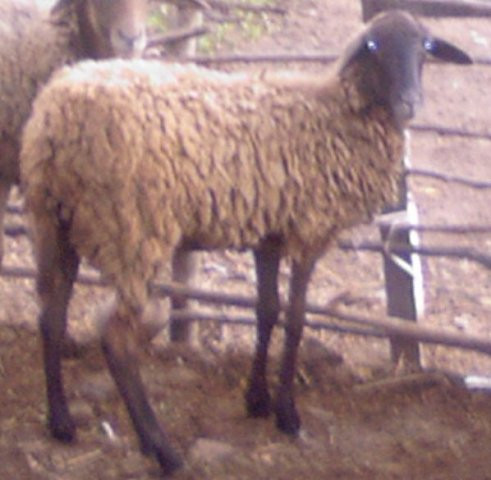
**Ram **(*Ovis aries*).

**Figure 6:**
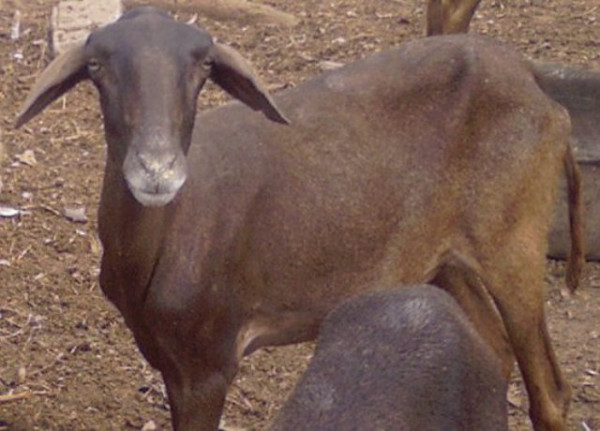
**Mele Goat **(*Capra hircus*).

**Figure 7:**
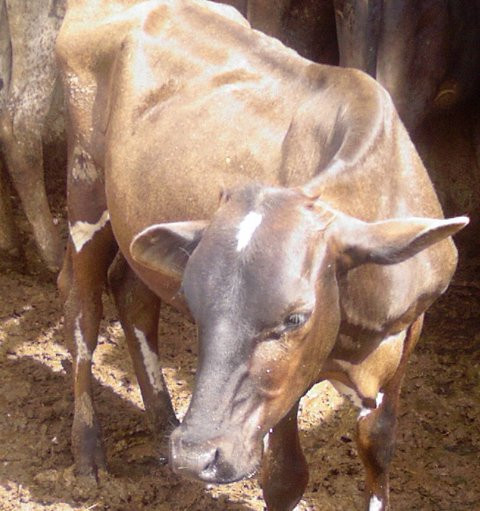
**Cattle **(*Bos taurus*).

**Figure 8:**
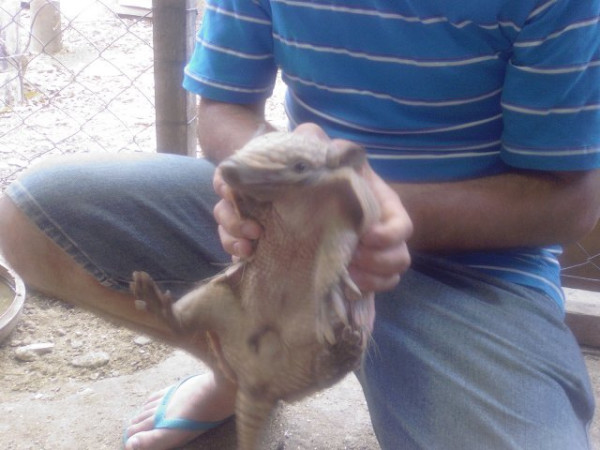
**Peba **(*Euphractus sexcinctus*).

**Figure 9:**
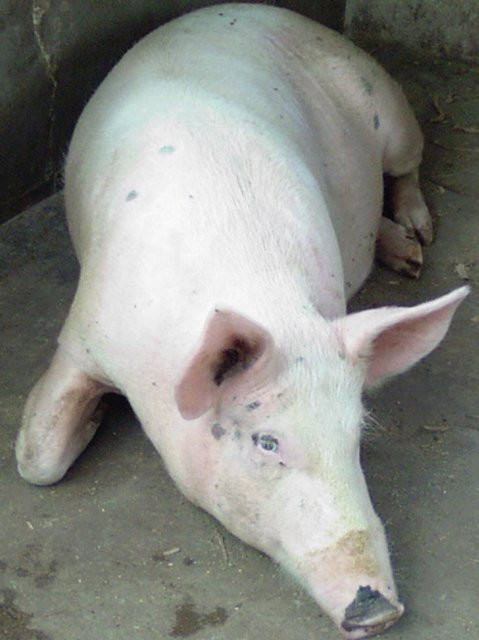
**Pig **(*Sus scrofa*).

**Figure 10:**
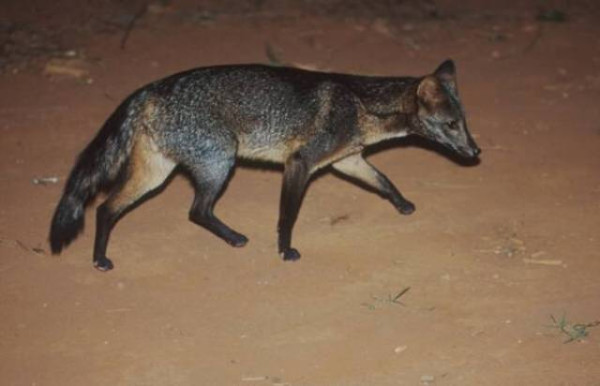
**Fox **(*Cerdocyon thous*). Font: IUCN [50].

**Figure 11:**
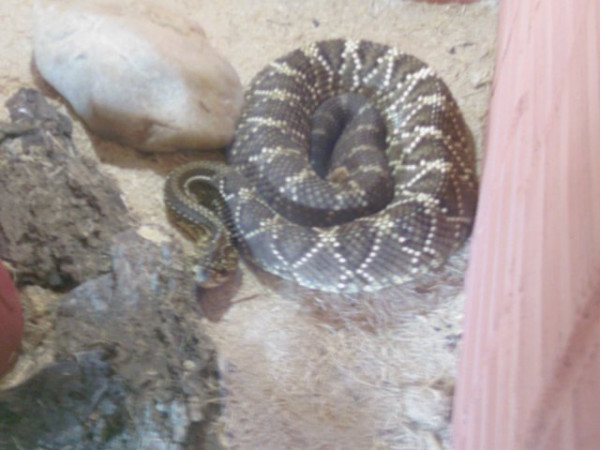
**Rattlesnake **(*Crotalus durissus*).

**Figure 12:**
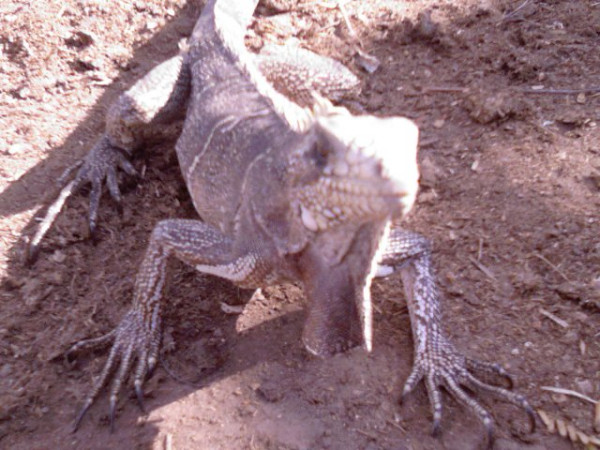
**Chameleon **(*Iguana iguana*).

**Figure 13:**
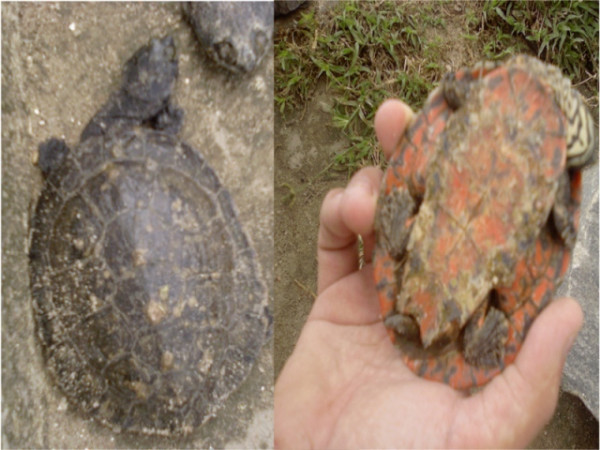
**Turtle of water **(*Phrynops spp*).

**Figure 14:**
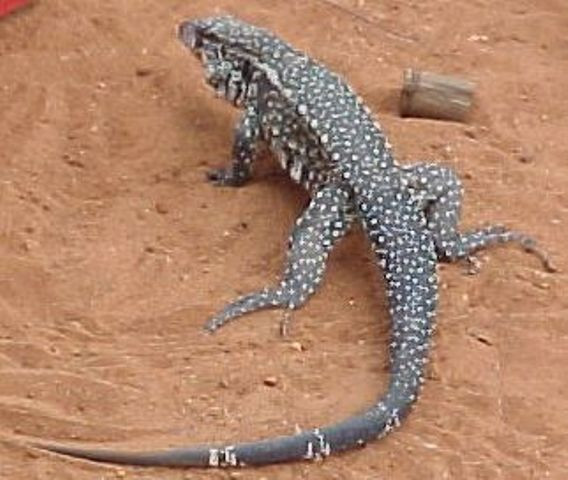
**Teju **(*Tupinambis merianae*).

**Figure 15:**
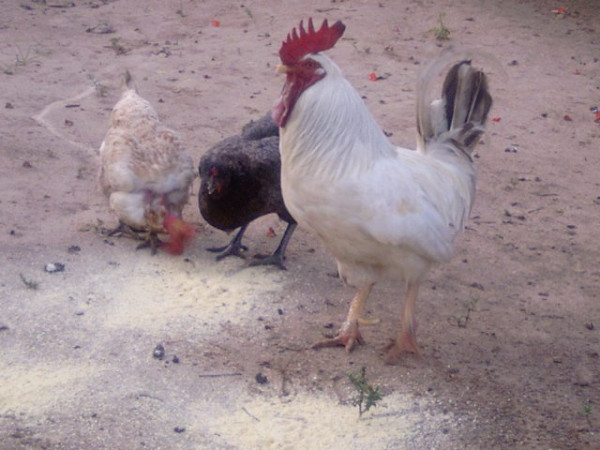
**Hen **(*Gallus domesticus*).

**Figure 16:**
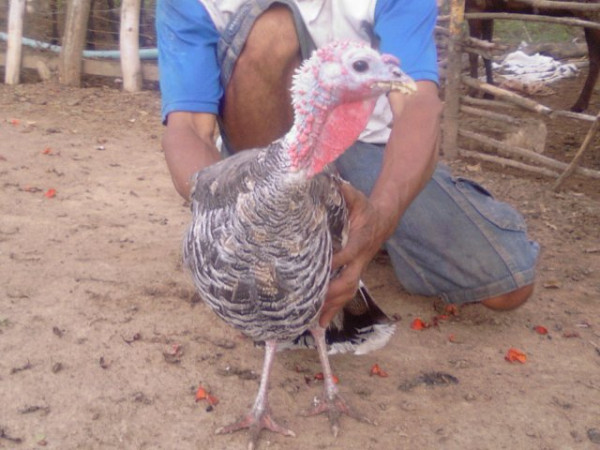
**Peru **(*Meleagris gallopavo*).

**Figure 17:**
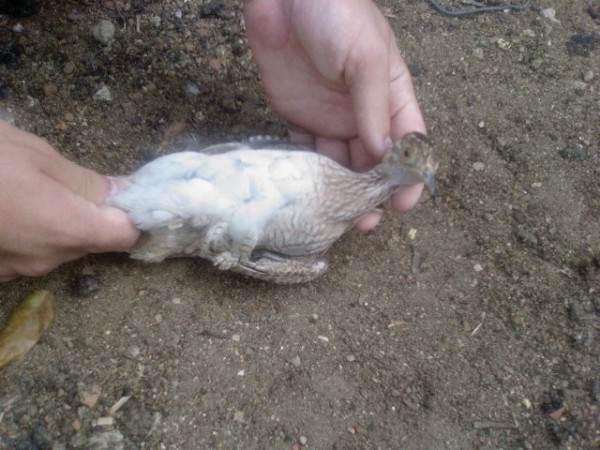
**Quail **(*Nothura maculosa cearensis*).

**Figure 18:**
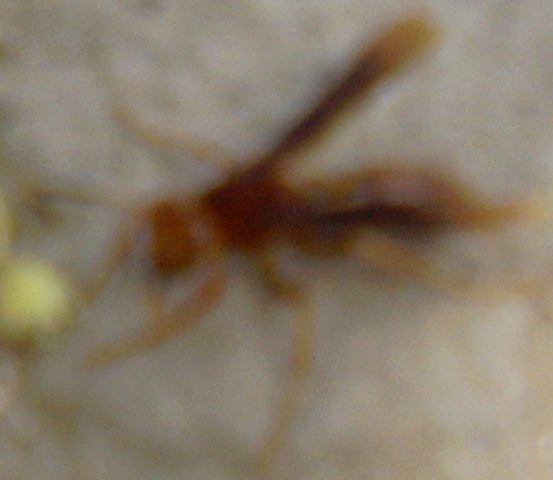
**Hornet-mestizo **(*Polistes canadensis*).

## References

[B1] Costa Neto EM, Oliveira MVM (2000). Cockroach is Good for Asthma: Zootherapeutic Practices in Northeastern Brazil. Human Ecology Review.

[B2] Mathias E, McCorkle CM (2004). Traditional livestock healers. Rev Sci Tech Off Int Epiz.

[B3] Campos MD'O (2002). Etnociência ou etnografia de saberes, técnicas e práticas?. Métodos de Coleta e Análise de Dados em Etnobiologia, Etnoecologia e Disciplinas Correlatas.

[B4] MARTIN GJ (1995). Ethnobotany: A Methods Manual.

[B5] Moura FBP (2002). Entre o peixe e o dendê: etnoecologia do povo dos Marimbús (Chapada Diamantina-BA). Dissertação de Doutorado.

[B6] Mathias E Introducing ethnoveterinary medicine. http://www.ethnovetweb.com/whatisevm.pdf.

[B7] Misra KK, Kumar KA (2004). Ethno-veterinary Practices Among the Konda Reddi of East Godavari District of Andhra Pradesh. Stud Tribes Tribals.

[B8] Costa Neto EM (2005). Animal-based medicines: biological prospection and the sustainable use of zootherapeutic resources. Anais da Academia Brasileira de Ciências.

[B9] Kakati LN, Ao B, Doulo V (2006). Indigenous Knowledge of Zootherapeutic Use of Vertebrate Origin by the Ao Tribe of Nagaland. J Hum Ecol.

[B10] Matekaire T, Bwakura TM (2004). Ethnoveterinary Medicine: A Potential Alternative to Orthodox Animal Health Delivery in Zimbabwe. Intern J Appl Res Vet Med.

[B11] Rodriguez JL, Bezerra CP, Magalhães CMGM, Telles GMVV, Silva JN, Carvalho MGRF, Travassos MSB, Maciel VS (2002). Atlas Escolar Paraíba.

[B12] Jacob MO, Farah KO, Ekaya WN (2004). Indigenous Knowledge: The Basis of The Maasai Ethnoveterinary Diagnostic Skills. J Hum Ecol.

[B13] ADH (2004). Atlas do Desenvolvimento Humano no Brasil.

[B14] IBGE – Instituto Brasileiro de Geografia e Estatística Canal Cidades@. http://www.ibge.gov.br/cidadesat/default.php.

[B15] EMBRAPA – Empresa Brasileira de Pesquisa Agropecuária Urbanização nos Municípios da Paraíba. http://www.urbanizacao.cnpm.embrapa.br/conteudo/uf/pb.html.

[B16] BRASIL (2005). Ministério de Minas e Energia. Secretaria de Geologia, Mineração e Transporte Mineral. Secretaria de Desenvolvimento Energético. Diagnóstico do Município de Cubati.

[B17] BRASIL Ministério da Integração Nacional: Rio São Francisco – Revitalização e Integração. http://www.integracao.gov.br/saofrancisco/projeto/risco.asp.

[B18] IBGE – Instituto Brasileiro de Geografia e Estatística População estimada do município de Cubati-PB. Canal Cidades@. http://www.ibge.gov.br/cidadesat/default.php.

[B19] IBGE – Instituto Brasileiro de Geografia e Estatística Lavoura Permanente do município de Cubati-PB. Canal Cidades@. http://www.ibge.gov.br/cidadesat/xtras/csv.php?tabela=lavperm&codmun=250500&nomemun=Cubati.

[B20] IBGE – Instituto Brasileiro de Geografia e Estatística Lavoura Temporária do município de Cubati-PB. Canal Cidades@. http://www.ibge.gov.br/cidadesat/xtras/csv.php?tabela=lavtemp&codmun=250500&nomemun=Cubati.

[B21] IBGE – Instituto Brasileiro de Geografia e Estatística Produção Pecuária no Município de Cubati-Pb. Canal Cidades@. http://www.ibge.gov.br/cidadesat/xtras/csv.php?tabela=prodpec&codmun=250500&nomemun=Cubati.

[B22] Marques JGW (1999). "Do canto bonito ao berro do bode": percepção do comportamento de vocalização em aves entre camponeses alagoanos. Revista de Etologia.

[B23] Andrade JN, Costa Neto EM (2006). O comércio de produtos zooterápicos na cidade de Feira de Santana, Bahia, Brasil. Sitientibus Série Ciências Biológicas (Etnobiologia).

[B24] Barboza RRD (2005). Os Moradores e as garças-vaqueiras (Bubulcus ibis) (LINNAEUS, 1758), dos municípios de Riachão do Bacamarte, Gurinhém e Cajá (Caldas Brandão)-PB: uma interpretação etnoornitológica e etnoecológica. Monografia.

[B25] Costa Neto EM (2000). Conhecimento e usos tradicionais de recursos faunísticos por uma comunidade afro-brasileira. Resultados preliminares. Interciencia.

[B26] Albuquerque HD, Albuquerque ICS, Monteiro JA, Barbosa AR, Sousa SM, Cavalcanti MLF (2004). Uso de plantas medicinais no tratamento de répteis em cativeiro: um estudo prelimina. Revista de Biologia e Ciências da Terra.

[B27] Alves AGC, Souto FJB, Leite AM (2002). Etnoecologia dos Cágados-d'água *Phrynops spp*. (Testudinomorpha: Chelidae) entre pescadores artesanais no açude Bodocongó, Campina Grande, Paraíba, Nordeste do Brasil. Sitientibus Série Ciências Biológicas.

[B28] IBAMA – Instituto Brasileiro de Meio Ambiente e Recursos Naturais Renováveis Lista das Aves da Paraíba. http://www.ibama.gov.br/cemave/download.php?id_download=53.

[B29] BRASIL (2002). Ministério da Saúde. Fundação Oswaldo Cruz: Animais Peçonhentos e Venenosos. http://www2.fiocruz.br/pdf/sinitox/serpentes.pdf.

[B30] Comitê Brasileiro de Registros Ornitológicos (CBRO) Lista das Aves do Brasil. http://www.cbro.org.br/CBRO/pdf/avesbrasil_jul2006.pdf.

[B31] Rodrigues MT Avaliação e Identificação de ações prioritárias para a conservação, utilização sustentável e repartição de benefícios da biodiversidade do bioma Caatinga – A fauna de répteis e anfíbios das caatingas. http://www.biodiversitas.org.br/caatinga/relatorios/repteis_anfibios.pdf.

[B32] Rodrigues MT (2003). Herpetofauna da Caatinga. Ecologia e Conservação da Caatinga.

[B33] Oliveira JA, Gonçalves PR, Bonvicino CR (2003). Mamíferos da Caatinga. Ecologia e Conservação da Caatinga.

[B34] Silva JMC, Souza MA, Bieber AGD, Carlos CJ (2003). Aves da Caatinga:*Status*, uso do habitat e sensitividade. Ecologia e Conservação da Caatinga.

[B35] Silveira L (1999). Ecologia e conservação dos mamíferos carnívoros do Parque Nacional das Emas, Goiás. Dissertação de doutorado.

[B36] Sousa MAN, Gonçalves MF (2004). Mastofauna terrestre de algumas áreas sobre influência da Linha de Transmissão (L.T.) 230 KV PE/PB, CIRCUITO 3. Revista de Biologia e Ciências da Terra.

[B37] Dawkins R (2001). O Gene Egoísta.

[B38] Araújo HFP, Lucena RFP, Mourão JS (2005). Prenúncio das chuvas pelas aves na percepção de moradores de comunidades rurais no município de Soledade-PB, Brasil. Interciencia.

[B39] Lima KEC, Vasconcelos SD (2006). Acidentes com animais peçonhentos: um estudo etnozoológico com agricultores de Tacaratu, sertão de Pernambuco. Sitientibus Série Ciências Biológicas.

[B40] Mourão JS, Araújo HFP, Almeida FS Ethnotaxonomy of mastofauna as practised by hunters of the municipality of Paulista, state of Paraíba-Brazil. Journal of Ethnobiology and Ethnomedicine.

[B41] BRASIL Ministério do Meio Ambiente: Lista Nacional das Espécies da Fauna Brasileira Ameaçadas de Extinção. http://www.mma.gov.br/port/sbf/fauna/index.cfm.

[B42] IUCN – The World Conservation Union White Oak Principles of Sustainable Use. http://iucn.org/themes/ssc/susg/docs/woprinciples.pdf.

[B43] Fagundes DJ, Taha MO (2004). Modelo animal de doença: critérios de escolha e espécies de animais de uso corrente. Acta Cirúrgica Brasileira.

[B44] USP – Universidade de São Paulo Novo modelo animal permite experiências com vacina contra febre reumática. http://www.usp.br/agen/repgs/2007/pags/007.htm.

[B45] Anderson WAD, Scotti TM (1976). Sinopse de Patologia.

[B46] Wattiaux MA Mastitis: The disease and its transmission. http://babcock.cals.wisc.edu/downloads/de/23.en.pdf.

[B47] Duarte L, Drago MC (2005). A raiva. Monografia.

[B48] Jardim GC, Pires PP, Mathias LA, Ribeiro OC, Kuchembuck MRG (2006). Diagnóstico sorológico da brucelose bovina em animais adultos vacinados com dose reduzida da cepa 19 de. Brucella abortus.

[B49] Almeida MB, Tortelli FP, Riet-Correa B, Ferreira JLM, Soares MP, Farias NAR, Riet-Correa F, Schild AL (2006). Tristeza parasitária bovina na região sul do Rio Grande do Sul: estudo retrospectivo de 1978–2005. Pesq Vet Bras.

[B50] IUCN – The World Conservation Union (2006). Canid Specialist Group – Crab-eating fox (*Cerdocyon thous*). http://www.canids.org/species/Cerdocyon_thous.htm.

[B51] Andrei E (1987). Compêndio Veterinário.

[B52] Kelly WR (1986). Diagnóstico Clínico Veterinário.

